# An Innovation in Cancer Nursing Education Across Europe: A Pilot Evaluation

**DOI:** 10.1007/s13187-024-02424-x

**Published:** 2024-04-09

**Authors:** Wendy McInally, Vanessa Taylor, Celia Diez de los Rios de la Serna, Virpi Sulosaari, Eugenia Trigoso, Sara Margarida Rodrigues Gomes, Ana Rita Cesario Dias, Silvija Piskorjanac, Mary Anne Tanay, Halldóra Hálfdánardóttir, Maura Dowling

**Affiliations:** 1 School of Health, Wellbeing and Social Care, The Open University, Milton Keynes, UK; 2 School of Health and Human Sciences, University of Huddersfield, England, UK; 3 PhD Programme, Faculty of Nursing, Bellvitge Campus, University of Barcelona (UB), 08907 Barcelona, Spain; 4 Health and Well-Being, Turku University of Applied Sciences, Turku, Finland; 5 Hospital University and Polytechnic LA FE, Valencia, Spain; 6 Nursing School of Coimbra (ESEnfC), Coimbra, Portugal; 7 Faculty of Dental Medicine, Health Centro Hospitalar E Universitário de Lisboa Central, Lisbon, Portugal; 8 University J.J. Strossmayer Osijek, Osijek, Croatia; 9 Florence Nightingale Faculty of Nursing, Midwifery and Palliative Care, King’s College London, London, UK; 10 Landspitali – The National University Hospital of Iceland, Reykjavik, Iceland; 11 School of Nursing and Midwifery, University of Galway, Galway, Ireland

**Keywords:** Cancer nursing, Oncology nursing, Nursing education

## Abstract

The European Oncology Nursing Society (EONS) is a pan-European not for profit society involving approximately 28,000 cancer nurses from 32 countries in the region. The European College of Cancer Nursing (ECCN) exists under the umbrella of EONS and was established in 2020 with a strategic priority to develop, promote and deliver educational opportunities for nurses across Europe. ECCN introduced a pilot on-line education programme for 20 nurses in January 2023. This study evaluated participating nurses’ views and experience of learning on the pilot programme. The study adopted a mixed method approach guided by the four levels of the Kirkpatrick theoretical framework. A dominant focus on qualitative data was used with supplementary quantitative data. The Standards for Reporting Qualitative Research (SRQR) was followed. Eleven nurses completed the pre-pilot online questionnaire (response rate 65%) and seven (*n* = 7) completed the post-pilot questionnaire (41% response rate). Five (*n* = 5) nurses participated in two focus group interviews. Data analysis resulted in the development of four overarching themes: A wider world of cancer nursing; Shapeless mentorship; Impact on Practice; Learning online and what now? On commencement of online education programmes, nurses value a structured timetable and support from nursing management to maximise engagement with the learning materials.

## Introduction

Cancer is a key priority worldwide for people affected by cancer [1]. Caring for people affected by cancer requires a range of specific knowledge, skills and experience in the delivery of complex care regimes within hospital and community settings [2].

Nursing plays a pivotal and often varied role in meeting the needs of people affected by cancer [3], and there is an expectation that the cancer workforce can meet these needs. As we strive to develop our future nursing workforce, it is imperative that all Higher Education Institutions (HEIs) embed cancer care within their pre-registration nursing programmes, so they have the knowledge and some experience once qualified [4]. In Europe, while there is standardization of education for entry-level (pre-registration) nursing [5], cancer content in entry-level curricula varies. Efforts to develop a common European cancer curriculum for pre-registration nurses are underway [6]. Similarly, following registration as a General Nurse, European nurses’ opportunities for specialist nursing (post-registration) education vary across countries [7].

It is important to recognise that cancer care is a highly specialised field of nursing practice, which requires a higher level of education, training, and competence, beyond undergraduate nursing education [7, 8]. This statement is reinforced by the European Code of Cancer Practice [9] p.35 in one of its ten key overarching rights, “You have a right to receive care from a specialised multidisciplinary team, ideally as part of a cancer care network”. Thus, it is essential to understand what is necessary within the cancer care pathway to enable Healthcare Professionals (HCPs) to work together holistically and to ensure this level of care is provided for all people affected by cancer [10, 11].

To support the cancer burden worldwide education and training of all HCPs including nurses, is crucial, especially in the current and predicted context of shortages in the healthcare workforce [12]. Embedding cancer care into entry-level (pre-registration) nursing programmes is recommended. Nurses caring for people with cancer consistently express the need for more education and training [13]. However, the opportunities for cancer nursing education and training vary, with 80% of Western and 27% of Eastern European countries implementing specialized training for oncology nurses [14]. The recent survey by EONS, including cancer nursing data from 38 of the 53 WHO European countries, found that as many as 17 (45%) countries do not provide university-level, specialist cancer nursing education that is nationally recognised. In addition, only 13 of the 38 countries (34%) offer Master programmes in cancer nursing and only 10 (29%) have professors in cancer nursing. This means that a large proportion of cancer nurses have limited access to specialist education and career opportunities in cancer care. This can have serious consequences for the future of cancer nursing and may impact care quality indicators such as patient safety [15].

The current shortage of nurses in Europe has various causes, including limited career prospects and education, low salaries, restricted participation in decision-making, migration and lack of professional standards and quality indicators [3]. WHO Europe state that the first step in tackling these challenges is to improve nursing education, at both pre- and post-registration level. Cancer nurses provide a 24-h care, and it is imperative that nurses provide the highest quality of cancer care. This requires accessible and quality education and training. While the Bologna Declarations (1999) have helped to harmonise undergraduate nursing education in Europe, major differences in specialist nursing education persist between countries [16]. Moreover, while great progress has been achieved in developing a common European Credit Transfer System to guide educational programs in Europe, it is increasingly challenging for nurses to find the time or funding to engage in continuing education, and there remains a wide variation in availability and access to continuing education for cancer nurses across Europe [17].

## Background

The European Oncology Nursing Society (EONS) is a pan-European not for profit society involving approximately 28,000 cancer nurses from 32 countries in the region. EONS provides leadership in all areas of cancer nursing, research, practice, continued professional development (CPD) through education, communication and advocacy across Europe. The EONS mission is to ensure that all people affected by cancer benefit from the care of highly educated, well-informed and competent cancer nurses, whether as an early career or experienced cancer nurse.

The European College of Cancer Nursing (ECCN) exists under the umbrella of EONS. ECCN was established in 2020 with a strategic priority to develop, promote and deliver educational opportunities for nurses across Europe in line with the EONS Education Framework (2022), which comprises eight modules focused on fundamental knowledge and skills required for post-registration nurses working with people affected by cancer. In particular, ECCN is committed to support nurses from the lower middle income European countries where education and need for learning is sparse [14].

Regardless of practice setting, all nurses will encounter people living with or beyond cancer within their area of practice, whether in a specialist or non-specialist environment. The ECCN offers a collegiate environment promoting and providing cancer education, professional development and networking opportunities for all nurses supporting people affected by cancer across Europe to enhance care.

Within the vision of EONS, the ECCN has three strategic priorities which underpin the college’s vision of advancing cancer nursing, as follows:

1. Developing, promoting and delivering educational opportunities guided by the EONS Education Framework which supports nurses at all stages of their career and levels of practice.
2. Fulfilling nurses’ professional development needs and career aspirations in a manner that is appropriate to their country or region of employment.
3. Supporting the EONS Working Groups of which there are five (Communication, Advocacy, Research, Education and Early career Nurse) to influence and shape cancer nursing education and continuing professional development policy and practice across Europe, building career and education pathways for the current and future cancer nursing workforce with the aim of improving care of all people affected by cancer.

Arising from ECCN’s vision, a learning pathway with three incremental levels was developed [7] illustrated in Table 1. This paper presents the evaluation of the first-level pilot offered to a sample of 20 nurses working in various cancer care settings across Europe. Because the college was a pilot initiative, it was agreed by the EONS board members that 20 nurses was an appropriate sample size.

**Table 1:** Summary of learning pathway aligned to EONS Cancer Nursing Education Framework

ECCN learning pathway	Target audience	Aims	Modules from EONS Cancer Nursing Education Framework
Minimum early career/registration-level nurse (early career nurse pathway)	Registered nurses who are new or inexperienced in caring for people affected by cancer and are seeking to develop their fundamental knowledge about cancer and its treatment, and principles of cancer care	To provide fundamental knowledge about cancer, the process of carcinogenesis and cancer treatment, and how this knowledge informs the delivery of person-centred holistic care for people affected by cancer	1 Risk reduction, early detection and health promotion in cancer care 2 Cancer pathophysiology and the principles of treatment decision-making 3 Cancer treatment, patient and occupational safety 6 Communication in cancer care

The ECCN’s pilot first-level pathway commenced in January 2023 and was accessed via an online learning platform. As the participating nurses were geographically dispersed across Europe the option of face-to-face was not feasible. The use of the Virtual Learning Environment (VLE) offers flexibility and is suited to all learners who are required to access the learning environment in ways that suit their work patterns, lifestyles and learning preferences, as well as the needs of future employers [18]. Positive experiences of the VLE suggest that it prepares nurses to be digitally aware and competent in using digital systems, required for both online learning and within contemporary health care practice where digital technologies like telehealth are being used more extensively [19, 20].

The college was supported by a Task group (TG), project administrator and a learning technologist throughout the development and delivery of the on-line materials. The learning materials which consisted of five learning blocks (Risk Reduction, Early Detection and Health, Higher Education England (HEE) modules 1 to 4, Nightingale Challenge and Safety webinars) aimed to enhance nurses’ practice by improving their understanding of cancer, cancer prevention, early diagnosis and treatment. The box illustrates the learning outcomes for each of the five learning blocks for the first level of the pathway. The pilot pathway was nine months in duration with pre-arranged synchronous online support sessions where all participating nurses could meet each other. Meetings were arranged for at least once per month with the timings altered from noon to early evening to accommodate nurses in practice. These sessions were not mandatory and attendance was minimal with two to four attending per session, mostly at noon. In addition, each participating nurse was assigned a mentor. All mentors were experienced cancer nurses and members of EONS. All mentors were orientated to their role on the pilot which was to support participating nurses’ learning on the pathway and in practice during the pathway. While mentorship was not compulsory, participants undertaking the pilot pathway were given the option, and all agreed to be assigned a mentor. Furthermore, online support by the TG was factored in at the beginning, middle and near the end. Participating nurses were encouraged to attend the online support by communicating dates in advance. In addition, within the Moodle environment there was an area for “chat” or any queries which was monitored weekly.

### Box

#### Learning Outcomes

(i) Discuss health promotion, early detection and prevention of cancer.
(ii) Demonstrate an understanding of the pathophysiology of cancer as a disease process.
(iii) Describe communication challenges in the cancer trajectory and strategies to therapeutic communication.
(iv) Outline treatments for cancer.
(v) Describe common adverse events from cancer treatments and principles of management.
(vi) Identify common oncological/haematological emergencies and outline their management.
(vii) Explain how distress is identified in a person with cancer and appropriate responses.
(viii) Describe how to recognize signs of compassion fatigue in oneself and others.

### Aims

This study aimed to evaluate participating nurses’ views and experience of learning on the ECCN pilot pathway. Specific objectives were as follows:

- To describe nurses’ views on and explore their experiences of learning.
- To identify enablers and barriers to learning on the pilot pathway.

### Methods

The study adopted a mixed method approach with a dominant focus on qualitative and supplementary quantitative data [21] and followed the Standards for Reporting Qualitative Research (SRQR) guideline [22]. The evaluation was guided by the first three levels of the Kirkpatrick (1967) theoretical framework; (reaction: how well did they like the programme; learning: what principles, facts and techniques were learned; behaviour what changes in behaviour resulted [23]. This framework is the most widely cited in educational evaluations [24] and has been used extensively in evaluating cancer education [25, 26]. Kirkpatrick proposed the levels as different but complementary. The first two levels of the framework guided the development of the post-pilot questionnaire. The third level was explored in the focus group interviews where nurses were questioned about perceived changes to their practice. Kirkpatrick (1967) frames the fourth level as “tangible results”, which was not possible to ascertain in the pilot. In future developments of the ECCN, the fourth level could be adopted by including line managers of participating nurses in the evaluation and their views on the clinical impact of the pilot ascertained.

### Setting and Study Population

Recruitment for the pilot was launched in September 2022 with an open call shared on the EONS website and social media. Using a scoring system, 20 nurses (from England, Ireland Romania, Croatia, Spain, Portugal and Greece) were chosen from a total of 33 applications.

## Data Collection

Qualitative data were collected through two virtual focus group on Microsoft Teams and interviews were recorded with nurses upon completion of the five learning blocks within the first level. Each focus group was approximately 60 min in duration. The interviews were facilitated by two authors (WM and MD), both experienced qualitative researchers.

Quantitative data were collected using an online questionnaire on QuestionPro® pre- and post-pilot. The pre-pilot questionnaire requested participants’ demographic, educational and work-related information. Participants’ comfort with their digital literacy skills was also measured pre- and post-pilot using a five-point Likert scale on 13 items related to their attitudes towards technology and technical dimensions of their digital literacy previously adapted by [27] from the Digital Literacy Scale [28].

Additional qualitative data were also collected through open questions on QuestionPro® asking participants about their motivation and workplace supports to undertake the pilot. The post-pilot questionnaire also asked participants to rank their experience of learning and satisfaction with the pilot using Likert scales. In addition, both pre- and post-questionnaires asked the nurses to rank how well prepared they thought they were to care for people with cancer, on a Likert scale of 1 (not at all prepared) to 10 (very well prepared) [29].

## Data Analysis

Two authors (WM and MD) undertook qualitative analysis guided by [30] reflexive approach. Reflective journaling and discussion between both authors were carried out to ensure critical reflection on the process of data collection and analysis. The transcripts were coded inductively, read and re-read to become familiar with the data. Initial codes were generated from the data and subsequently organised and reorganised, searching for themes and sub-themes. Themes were reviewed through a deductive re-analysis process by the wider project team; themes which lacked sufficient data were discarded. This shift from coding should maintain complexity and depth, which was created through exploratory coding while also reducing the amount of data.

Quantitative data were descriptively analysed from data obtained on the pre- and post-pilot online questionnaire. The final stage of analysis included discussion of both qualitative and quantitative findings to reach an agreed interpretation of participants’ experience of learning on the pilot pathway.

## Ethical Considerations

Ethical approval to undertake the evaluation was granted by the Research Ethics Committee of the University of Galway, Ireland (Ref: 2022.11.005). All participants received information explaining the purpose of the study, pseudonymization of data, and requested to complete an online consent using QuestionPro® providing their email address if they wished to participate. Following completion of the online consent, participants were emailed the link to the pre- and post-pilot questionnaires and invited to participate in the focus groups.

## Findings

Seventeen participants consented to take part in the programme evaluation (response rate of 85%). However, following two reminders, 11 completed the pre-pilot online questionnaire (response rate 65%) and seven (*n* = 7) completed the post-pilot questionnaire (41% response rate) (Table 2 and 3). There was one male participant, all were aged between 25 and 40 years, and had little or no education and training in cancer care post-registration. Participants’ experience in cancer care ranged from 1 to 15 years with all having some experience of online learning.

**Table 2:** Participant characteristics

	*n*	%
Gender		
Female	11	
Male	1	
Age		
Mean age in years, age range	34.8 (25–57)	
Highest education		
Masters	3	25
Postgraduate diploma	1	8.33
Bachelor’s degree	5	41.67
Diploma	3	25
Most recent (last) qualification		
Less than 5 years ago	9	75
Between 6 and 10 years	1	8.33
More than 11 years ago	2	16.67
A post-registration qualification in oncology nursing		
No		75
Yes		25
Cancer focused training or education courses taken in the previous 6 months		
No	9	75
Yes (1. Chemotherapy administration; 2. Immunotherapy; 2; ABC4 Nurses programme)	3	25
Current oncology work setting		
Surgical oncology	0	
Oncology-haematology day unit	4	30.77
Medical-oncology unit	5	38.46
Radiotherapy Unit	1	7.69
Community	0	
Mixed medical unit	0	
Haematology unit	1	7.69
Other (acute oncology)	1	7.69
Country of residence		
England	3	25
Spain	3	25
Greece	2	16.6
Croatia	2	16.6
Wales	1	8.3
Ireland	1	8.3
Years qualified as a nurse		
Mean, range	9 (1–24)	
How well prepared do you think you are to care for people affected by cancer? (1–100)		
Average score	58.08

**Table 3:** Post-pilot questionnaire, *n* = 7 respondents

	*n*	%
The length of the pilot was…		
Too long	0	
Just right	7	100
Too short	0	
The speed of the presentations/videos was…		
Too fast	0	
Just right	6	85.71
Too slow	1	14.29

Five (*n* = 5) nurses participated in two focus group interviews. All were employed in either adult, or children and young peoples’ cancer services in England, Spain and Croatia.

Data analysis resulted in the development of four overarching themes from twenty two sub-themes.

### Theme 1: A Wider World of Cancer Nursing

This theme describes participants’ feelings and views following their exposure on the pilot to cancer care in other practice centres across Europe. The experience provided them with an insight into “a room with a view” to a broader cancer nursing world, with opportunities and possibilities. It also enabled them to recognise that practice challenges and barriers they have experienced in their own countries also exist in other countries; and learned about potential solutions in the process. As one nurse explained: * “ I felt a bit stuck about what I could do and this (the pilot programme) gave me different opportunities that I have taken and I’m trying to start a new project at the hospital now after doing this (pilot programme) so this is gratifying and it gave me a bit more of a purpose or took me out of that feeling of being, feeling stuck as a nurse, as an oncology nurse, so it was a good experience, made me take my career to another step. “ Listening to other nurses, things that can be done, that I was unaware off that existed or that these things could be done[…]”* [P1:FG1]. 

This view was supported by another participant: * “[…] the thing that I noticed and I may be wrong, I think there were a couple of UK speakers from [names area] and I recognise that although it’s all hospitals from different countries around Europe the problems and the situations everyone faces are very similar, even though the healthcare systems are set up differently” * [P3:FG1]. 

In addition, most expressed that the opportunity to be part of this pilot was welcomed and they *“[…] found it very interesting, and very informative”* [P45:FG2].

### Theme 2: Shapeless Mentorship

Participants were provided with the contact details of their named mentor and mentors were encouraged to contact their mentee. However, little or no mentoring ensued following the initial contact between mentors and mentee. Participants were unclear on what the mentorship process entailed.

Some disappointment in the mentorship experience * “For me, I didn’t know what I was expecting. In the beginning, I introduced myself with an email and she told me to ask her whatever I needed but she wasn’t going to be on top of me all the time”* [P1:FG1]. *“It wasn’t clear what their role should be so I don’t know how much input they needed to our overall learning and I think that I’m sure they’d be there if you needed them, could email if I had any problems. I sent one email towards the end saying I’m not sure whether I have completed it [pilot program] correctly and they posted me in the right direction. So I think, as long as it’s set out what support they need, what to contact them if you needed….I didn’t need that sort of input but I presume they would be there if I did….I didn’t know whether we needed to do anything for the mentorship program”* [P3:FG1]. Some disappointment in the mentorship experience was also shared.

*“I honestly expected more. As it was introduced as a college as not a course I expected that my mentor would have been involved a lot more. I think I had one meeting with my mentor in the beginning and afterwards when I wanted to discuss about later meetings I didn’t get a last answer to it. I didn’t want to be pushy. So I honestly thought it would be like where somebody would guide me and we potentially would do something together as a team"* [P2:FG1]. Nonetheless, the potential of a mentoring relationship beyond its intention was revealed in the experience of one participant who reached out to their mentor for guidance in introducing a service initiative at their hospital. The mentor linked with another colleague in EONS who together, supported the nurse in introducing the initiative.* “So, I got introduced this this [the pilot programme] but we haven’t really been doing much for this course, but more from what I want to do from the outside, which is related to this as well. So it worked out for me because I didn’t know what I could use from them [mentor]….she was available to me when I had that doubt, what can I do here in [names country], I want to move on with this idea and she has been very helpful and we keep in touch with updates. For me, it worked out for what I wanted and what I needed”* [P1:FG1].

### Theme 3: Impact on Practice

This theme reflects participants’ views on the changes perceived in their practice. These varied from a better understanding of safety and cancer treatments to a deeper confidence and appreciation of holistic care. * “[…] the thing that I really got was me as an individual, just to know what to look after in my patients, look at the more holistically and to be aware of their psychosocial needs and not just their physical needs and just to take the time for being there with them and to talk with them and to learn about the right tools and how to address their needs [pauses] it’s really nice when you get to know proper tools to use with your patients”* (P2:FG1). *“I think around the basic stuff of cancer treatments and I explain a lot about this to patients at my hospital”* [P3:FG1]. *“I took some ideas from the safety information, the idea of the red jacket, or green one or whatever so we don’t get distracted giving medication […] I brought this idea to my superiors which they thought was good and at least to try, especially knowing that it has worked previously in different hospitals and it’s actually useful. And the other one as well related to patient safety” * [P1:FG1]. *“The course gives you the confidence to speak out about practice”* [P4:FG2]. 

The post-pilot questionnaire responses support this finding. The items “The pilot project was a worthwhile use of my time” and “I would recommend this pilot project to my co-workers” were ranked 4.67 and 4.57 out of a score of 5, respectively. Moreover, participants’ scoring for how well prepared they were to care for people affected by cancer increased from an average of 58.08 pre-pilot to 73.88 post-pilot (Table 4).

**Table 4:** Post-pilot questionnaire, *n* = 7 respondents

	Average score
Overall learning experience on the pilot program (Max 5)	
I was clear about the purpose of the pilot education project before I attended	4.43
This pilot project met my expectations for learning	4
The pilot project was a worthwhile use of my time	4.67
I believe that the pilot project has helped me in my work	4.43
I would recommend this pilot project to my co-workers	4.57
The learning materials were interesting	4.35
The learning materials made me feel engaged with the pilot project	3.57
The learning materials provided up-to-date/trustworthy information	3.86
The learning materials used easy-to-understand language	4.14
Online access to learning materials was useful	4.14
Online access to learning materials was easy	4.14
The learning blocks used appropriate practical examples to support learning (e.g. examples from practice)	3.71
The speakers/presenters were knowledgeable about the topic/s presented	4.43
The learning materials were clear and organised	3.43
Overall satisfaction with learning	
The contents of this pilot pathway have increased my knowledge of health promotion and prevention of cancer	4.43
The contents of this pilot pathway have increased my knowledge of the pathophysiology of cancer	4.57
The contents of this pilot pathway have increased my knowledge of the approaches used to diagnose and stage cancer	4.29
The contents of this pilot pathway have increased my knowledge of occupational safety in cancer	4.29
The contents of this pilot pathway have increased my understanding of communication challenges in cancer and strategies that I can use for therapeutic communication	4.29
I believe that the contents of this pilot pathway have increased my ability to recognise distress in a person with cancer and I know what appropriate response to make	4.29
I believe that the contents of this pilot pathway have increased my ability to recognise compassion fatigue in myself and others	4.29
The contents of this pilot pathway have increased my knowledge of treatments for cancer	4.43
The contents of this pilot pathway have increased my knowledge of common adverse events from cancer and how to manage these adverse events	4.43
The contents of this pilot pathway have increased my understanding of how to recognise oncological emergencies and how they are managed	4.43
The contents of this pilot pathway have increased my knowledge of early breast cancer	3.86
How well prepared do you think you are to care for people affected by cancer? (1–100)	
Average score	73.88
How committed are you to apply what you have learnt on the ECCN level 1 pilot pathway to your work? (1–100)	
Average score	91.67

### Theme 4: Learning Online and What Now?

Participants’ responses to the pre- and post-pilot digital literacy indicates their high level of digital literacy (mean scale score of 3.9 pre-pilot and 3.97 post-pilot). This was supported in the qualitative findings with no participant expressing any issues with using online learning resources. However, participants did share views with challenges experienced using the programme’s materials and delivery and shared their views on future directions for the college. *“Some of the recorded sessions could be re-recorded […] some of them are very long and you cannot hear what they are saying and they are interrupted by the questions from the people that were there and the people giving them that are not English speakers as well, it’s way more difficult for us to understand. I had to pay more attention to listen. It was very tiring” * [P1:FG1]. *“The thing that I found was missing, I would appreciate if we had one or two sessions [live] like maybe per module, that would be a good thing for me. If I had some misunderstanding or something wasn’t clear for me because some of the health systems in Europe are completely different, and just for some kind of a clarification, I would appreciate that […] I would prefer if recorded sessions came with a transcript”* [P2:FG1].

Issues with the webinar recordings were highlighted and the challenges in understanding speakers whose first language is not English.

Suggestions for the future role of the ECCN included the need for some form of assessment to promote engagement and feedback and synchronous sessions. *“It should be a college, it’s not just a course, should be more part of a project, do some kind of assignments, more interactive”* [P2:FG1]. *“Maybe it would be easier for us, to create a kind of small classroom, just to get to know each other and maybe to exchange our opinions…could be a useful platform in networking”* [P2:FG1]. *“But I do believe that your focus session should be part of the course that you have to set a time and a date, be it a weekend or an evening or something, where you have to attend to make people become a part of the team of learning”* [P4:FG2].

In addition, participants suggested that a structured timetable should be provided at the beginning: * “Work is so busy and “there is no time to learn” [P5:FG2]. therefore more structure and timeline at beginning required”*[P5:FG2].

The need for mandatory attendance at synchronous sessions was also considered essential to promoting participants’ learning experience. * “Have a prerequisite at the beginning of the course that you have to attend at least two or three to pass the course. […] Make some of the sessions mandatory […] a couple of live sessions as well because it’s hard to follow something and if you’ve got a question […] and people could suggest what sessions they would find helpful […] I think assessment, it’s more formal; you’re doing a piece of work as opposed to watching video after video”* [P3:FG1]. *“Maybe make one or two mandatory sessions [to attend] and even attending one, you might see that it’s actually helpful and you might want to join the rest” * [P1:FG1]. 

These findings are supported by the post-pilot questionnaire responses (Table 4). The items with the lowest rating (0–5) included “The learning materials made me feel engaged with the pilot project” (average score 3.57), “The learning materials were clear and organised” (average score 3.43), and “The learning materials made me feel engaged with the pilot project” (average score 3.57).

## Discussion

Online education has the potential to enhance cancer nurses’ access to education that supports professional development but poses challenges, including some learners’ difficulties with webinar speed and length (3). Also, educational opportunities to develop their knowledge about cancer and cancer nursing in countries where limited education exists. This will be addressed with the TG and new materials are being developed taking into consideration the speed, length and language. For example, shortening the hourly e-sessions to a maximum of 20 min and allowing some extra time for reflection and/or questions at the end to solidify the learning. In addition, ensuring that there are support sessions built into the timeline will also open up opportunities to connect with nurses delivering care to people affected by cancer in other countries to compare and contrast the challenges and sharing good practice. Our findings support this, highlighting issues with the quality of webinar recordings and understanding speakers whose first language is not English. For example, the Nightingale Challenges were not all developed and delivered by nurses from the UK where English is the first language. Moreover, our findings highlight the importance of interaction for engagement in online learning with participants suggesting synchronous learning as an approach to improve their online learning experience. This is supported by a qualitative study exploring rural nurses’ experiences of continuing professional development (CPD) in Australia, where key findings included the importance of addressing a range of learning styles, including feedback and opportunities for peer interaction [31]. In addition, using digital tools that support learner interaction is a key element of well-designed online programmes [32].

Despite the challenges with on-line learning, a recent systematic review (*n* = 15 studies) reported that irrespective of approach, learning activities and country, e-learning is an effective approach for nurses’ assimilation of theory and practice when compared to traditional learning approaches [33], and an ideal platform to increase the availability of education for specialist cancer nursing, as outlined in the aims of the RECaN (Recognition of European Cancer Nursing) project [15] where the overall goal is to increase recognition of the value and contribution of cancer nursing across Europe.

However, our findings have revealed limited representation from across Europe especially from the lower middle income European countries where education and need for learning is sparse which we know from the European Cancer Nursing Index (3). While it is unclear why few nurses from these countries applied for the pilot, a recent integrative review of mobile-social learning for CPD in low- and middle-income countries reports high acceptability for using digital platforms [34]. Furthermore, the focus group discussions did highlight limited support from line managers and employers. In addition, responses to an open question on the pre-pilot questionnaire revealed that nine participants did not receive support, such as some time off work to engage with the pilot’s learning materials, from their managers. Support should be made available to learners as evidence suggests leadership and support from key stakeholders are essential for HCPs continuing professional development particularly in lower middle-income countries including for services to benefit from the specialist knowledge development of their employees and for the delivery of enhanced patient care [35].

However, whilst cancer education in nursing programmes both pre- and post-registration level is a priority [4, 36], online learning facilitates flexibility in learning and can be undertaken in any location at any time. Mastering digital learning can also help prepare nurses to become proficient digital users and learners which is a key skill required for modern health care settings and employment in today’s society. Face-to-face learning is often cited as being preferable to online learning and can be less time consuming for instructors [37]. However, where this is not possible other avenues of the virtual learning environment (VLE) can be utilised. It is the intent of the college moving forward to have the European School of Oncology (ESO) and the EONS Masterclass embedded within the third level of the pathway where nurses spend a week learning face to face with other HCPs as well as nurses within a European country. These Masterclasses are open every year for approximately 30 nurses from across Europe to attend for 1 week’s face-to-face teaching.

Mentorship is also a requirement to support nurses in their professional development and careers and is important to fostering learner connectivity during online education [38]. The nurses however did not engage fully with the mentorship as it was out with the college and the did not see the connection. The key role for the future of mentorship is to align to the college where there will be support throughout the learning process for both mentors and mentees. The mentorship will be embedded into the VLE and support through workshops for both the mentors and mentees will be provided to enrich the experience for both. This will also support the engagement from both parties and the need for this partnership to work. Mentoring through the college with the support of EONS specialist cancer nurses supports the relationship between early career and more experienced nurses and is important for developing the next generation of cancer nurses [39].

Nurses in our study suggested that attendance at pre-arranged support sessions should be mandatory. Providing an introductory session where employers/line managers are invited to attend with the learner they are supporting and also a final evaluation meeting to discuss how the learning is informing clinical practice would be beneficial as part of the college. The need for a structure and an agreed timetable on commencement of a learning block is imperative to the overall commitment of the nurses but also with their manager. Setting the commitment at the onset would support the overall engagement. While facilitated peer group sessions were integrated into the pilot, attendance was poor. The nurses who did attend suggested that this was mainly due to work demands and not having the support of their employer. A requirement to attend a minimum number of pre-arranged synchronous sessions would encourage peer support. It is known that peer interaction online promotes learner engagement and a sense of belonging [40]. Also, having academic or ECCN recognition for their learning instead of a certificate from EONS would benefit them for professional development within their clinical roles.

## Strengths and Limitations

This evaluation has highlighted strengths and limitations of a pilot learning program for European oncology nurses. The pilot was evaluated drawing on Kirkpatrick’s framework. Kirkpatrick’s first two levels (reaction and learning) have been revealed in participants’ experiences and views on the programme and what they learned [23]. In addition, in an attempt to address participants’ learning support needs, queries posted after completion of each learning block were responded to through use of the chat box embedded in the online tools. Kirkpatrick’s third and fourth levels (behaviour and results) are more challenging to establish [25], especially in the context of different cancer care settings and various levels of cancer experience. However, some comments suggest that participants were attempting to use their learning to introduce changes to their clinical practice. A limitation was the voluntary sample, and small number of nurses who applied from specific European countries where the evidence suggests that education and training is absent. Also, not all participants engaged in the learning blocks to completion. Going forward, proactive regular engagement by the programme team with participants and managers may encourage and support opportunities for learning to be applied in participants practice being explored.

## Conclusions

This evaluation, focused on a pilot programme for early career nurses, will inform strategic developments in continuing education for cancer nurses across Europe, supported by EONS.

Cancer nursing across Europe is a unique speciality and one that has seen many changes over the past 40 years [41]. As was discussed throughout the limited availability of cancer education in some EU countries, this programme illustrates the potential to deliver learning, improving access and enhancing the knowledge and capabilities of nurses providing care to people affected by cancer across Europe, going some way towards meeting the WHO priorities. The education and training of nurses specialising within cancer care has become paramount in these constantly evolving specialities. Working within specialist or non-specialist settings where people with cancer are being cared for, the environments are facing fresh challenges ahead, especially as one in two people will be diagnosed with cancer and the survival rates continue to grow [7]. As these improvements develop, ongoing education and training is essential and necessary to assist in the effective delivery of prevention, treatment and supportive care regimes. Post-pandemic, although online learning is favoured by many organisations and institutions, a blended learning approach is often preferred by participants. This was expressed by the nurses and they would have appreciated more structured built in time for online support during the first level or face to face had resources allowed. Moving forward, this evaluation will support the development of the college and the needs of nurses from across Europe.
